# Bavachalcone Enhances ROR*α* Expression, Controls Bmal1 Circadian Transcription, and Depresses Cellular Senescence in Human Endothelial Cells

**DOI:** 10.1155/2015/920431

**Published:** 2015-06-23

**Authors:** Yanqi Dang, Shuang Ling, Jing Ma, Rongzhen Ni, Jin-Wen Xu

**Affiliations:** Murad Research Institute for Modernized Chinese Medicine, Shanghai University of Traditional Chinese Medicine, Shanghai 201203, China

## Abstract

The circadian clock regulates many aspects of (patho)physiology in the central nervous system and in the peripheral tissues. RAR-related orphan receptor *α* (ROR*α*), an orphan nuclear receptor, is involved in circadian rhythm regulation, including regulation of cardiovascular function. Bavachalcone, a prenylchalcone, is a major bioactive chalcone isolated from *Psoralea corylifolia*. This natural ingredient activated ROR*α*1 luciferase reporter activity on drug screening. In addition, bavachalcone induced ROR*α*1 expression in mRNA and protein levels in a dose-dependent manner and enhanced the circadian amplitude of Bmal1 mRNA expression after serum shock. Moreover, bavachalcone suppressed senescence in human endothelial cells and mRNA expression of p16^ink4a^ (a marker of replicative senescence) and IL-1*α* (a proinflammatory cytokine of the senescence-associated secretory phenotype). These inhibitory effects were partially reversed by the ROR*α* inhibitor VPR-66. Our results demonstrate that bavachalcone, as a natural medicine ingredient, has a pharmacological function in regulating ROR*α*1.

## 1. Introduction

The circadian clock not only controls the central nervous system, but also regulates many aspects of (patho)physiology in the peripheral tissues, including cardiovascular function. ROR*α*, an orphan nuclear receptor, is involved in the circadian rhythm regulation. ROR*α*1 and ROR*α*4, not ROR*α*2 and ROR*α*3, are the predominant forms of ROR*α* expression in vascular endothelial cells, smooth muscle cells, and fibroblasts [1, 2]. ROR*α* directly activates transcription of the circadian gene Bmal1 through conserved ROR*α* response elements [3, 4]. Homozygous Rora (sg/sg) mutant mice exhibited an enhanced susceptibility to atherosclerosis and hypoalphalipoproteinemia [5]. In addition, ROR*α* has been identified as a regulator of human apo A-1, A-5, and C-3 gene expression [5–7]. ROR*α* not only suppressed TNF-*α*-induced VCAM-1 and ICAM-1 expression in human endothelial cells [8], but also reduced oxidative stress through the induction of SOD2 and GPx1 expression [9]. Moreover, reduced circadian clock expression was closely associated with age in genetic hypertensive rats [10].

Bavachalcone, a prenylchalcone, is a major bioactive chalcone isolated from* Psoralea corylifolia*; it exhibits many biological activities. For example, Lee et al. reported dose-dependent reduction of inducible nitric oxide synthase activity in activated microglial cells treated with bavachalcone [11]. In osteoclasts treated with bavachalcone, Park et al. observed reduced activation of MEK, ERK, and Akt and suppression of osteoclast differentiation through NFATc1 [12]. In addition, a strong inhibitory effect of bavachalcone against 2 important isoforms of UDP-glucuronosyltransferases (UGT), UGT1A1, and UGT1A7, was observed [13]. In a previous study, we found that bavachalcone activates AMPK kinase activity, promotes the expression of MnSOD, and reduces mitochondrial superoxide anion [14]. On drug screening, bavachalcone enhances ROR*α*1 expression. Therefore, this study investigated the pharmacological effects of bavachalcone on ROR*α*1.

## 2. Materials and Methods

### 2.1. Cell Cultures

Human umbilical vein endothelial cells (HUVECs) (CRL-1730) were ordered from ATCC (Manassas, USA), and human embryonic kidney 293 (HEK-293) cells were purchased from the Institute of the Chinese Academy of Medical Sciences Cell Bank (Shanghai, China). HUVECs were maintained in a 37°C incubator supplemented with 5% CO_2_ in Dulbecco's modified Eagle's medium (DMEM) containing 10% fetal bovine serum. The medium was changed every 2 days and the cells were passaged with trypsin-EDTA. The population-doubling level (PDL) was calculated after each passage as PDL = (log⁡10*Y* − log⁡10*X*)/log⁡10^2^, where *Y* is the number of cells counted at the end of the passage and *X* is the number of cells seeded. Cumulative population doubling was calculated as the sum of all PDL the changes [15].

### 2.2. Serum Shock Procedures

Serum shock was performed as follows: approximately 1 × 10^6^ cells per 60-mm petri dish were plated 1 day prior to the experiment so that the cells would be approximately 80% confluent on the day of the experiment. The medium was exchanged with 0.3% serum DMEM medium for 16 h. At time *t* = 0, the medium was exchanged with serum-rich DMEM containing 50% new born bovine serum with or without 20 *μ*mol/L bavachalcone; after 2 h, this medium was replaced with 0.3% serum DMEM with or without 20 *μ*mol/L bavachalcone. At the indicated times, the petri dishes were washed twice with ice-cold phosphate buffered saline (PBS) and frozen on a liquid nitrogen layer or stored at −70°C until the extraction of whole cell RNA.

### 2.3. ROR*α*1 Reporter Luciferase Plasmid Construct and Assay

The thymidine kinase (−83 to +91) plus 3× RORE [TCG ACT CGT ATA ACT AGG TCA AGC GCT G] sequence was generated by inserting the corresponding annealed oligonucleotides into the Luc pGL3-basic plasmid. HEK293 cells were cultured in 24-well plates at a density of 2 × 10^5^ cells/well. When the cell density reached 70%–80%, the cells were used for transfection. In each well, 0.8 *μ*g of a reporter vector of the pGL3 ROR*α* reporter firefly luciferase gene and 0.016 *μ*g of a reporter pRL-SV40 plasmid of the Renilla luciferase gene as normalization control were transfected and diluted with the FuGENE HD transfection reagent in the Opti-MEM transfection medium. The medium was replaced with normal DMEM 6 h after transfection. After incubation with bavachalcone (Shanghai Yuanye Bio-Technology Co., Ltd., Shanghai, China) for 16 h, the activity of the luciferase reporter gene was assayed using the dual-luciferase reporter 1000 assay system and detected using a Varioskan Flash microplate spectrophotometer (Thermo Scientific, USA).

### 2.4. Quantitative Real-Time Polymerase Chain Reaction

Total RNA was extracted using TRIzol (Life Technologies, Carlsbad, USA) according to the manufacturer's instructions. Real-time polymerase chain reaction (PCR) amplification and detection were performed using the SYBR Green qPCR SuperMix-UDG with ROX (Life Technologies) in a fluorescence thermal cycler (StepOne real-time PCR system, Life Technologies) according to the manufacturer's protocol. Gene expression was normalized using GAPDH as a reference gene. The primers used in our study are as follows: ROR*α*, forward: 5′-CAG GCT TCT TTC CCT ACT GTT CGT-3′, reverse: 5′-CCG CTG CTT GTT TTG ATA GTT CTC-3′; BMAL1, forward: 5′-CAA TCC ATA CAC AGA AGC AAA CTA C-3′, reverse: 5′-ACA TCC TAC GAC AAA CAA AAA TCC-3′; P16-INK4A (CDKN2A), forward: 5′-TTT TCA CTG TGT TGG AGT TTT CTG G-3′, reverse: 5′-TGA GCT TTG GTT CTG CCA TTT G-3′; IL-1*α*, forward: 5′-GCC CAA GAT GAA GAC CAA CCA GT-3′, reverse 5′-CCG TGA GTT TCC CAG AAG AAG AGG-3′; GAPDH, forward: 5′-CGC TGA GTA CGT CGT GGA GTC-3′, reverse: 5′-GCT GAT GAT CTT GAG GCT GTT GTC-3′; 18S rRNA, forward: 5′-AGG TCT GTG ATG CCC TTA GAT GTC-3′, reverse: 5′-TCC TCG TTC ATG GGG AAT AAT T-3′.

### 2.5. Western Blot

After treatment, the cells were centrifuged and lysed in Triton/NP-40 lysis buffer containing 0.5% Triton X-100, 0.5% Nonidet P-40, 10 mmol/L Tris (pH 7.5), 2.5 mmol/L KCL, 150 mmol/L NaCl, 20 mmol/L *β*-glycerolphosphate, 50 mmol/L NaF, and 1 mmol/L Na_3_VO_4_, sonicated by JY92-2D ultrasonic homogenizer (NingBo Scientz Biotechnology Co., Ltd, Zhejiang, China) and centrifuged for 10 min at 10000 g. The supernatant was employed for protein concentration measurement using a protein assay kit (Bio-Rad, Hercules, CA, USA), and 30 *μ*g of protein was separated through sodium dodecyl sulfate-polyacrylamide gel electrophoresis and transfered to nitrocellulose membranes (Pall China, Shanghai, China). The membranes were blocked overnight with 5% nonfat dried milk in a buffer containing 140 mmol/L NaCl, 20 mmol/L Tris-HCl (pH 7.5), and 0.1% Tween 20 and incubated with the following primary antibodies: ROR*α* rabbit polyclonal antibody (ab60134, Abcam, USA) and anti-GAPDH monoclonal mouse antibody (KangChen Bio-tech Inc., Shanghai, China). Finally, the membranes were incubated with a horseradish peroxidase (HRP) conjugated secondary antibody, at 4°C with gentle shaking overnight. The membranes were exposed to high-performance autoradiography film (Fuji film Co., Tokyo, Japan) and visualized using the ECL immobilon western chemiluminescent HRP substrate (WBKLS0500, Millipore, USA). Quantitative analysis was performed using Quantity One software (Bio-Rad Laboratories). Western blot experiments were performed in triplicate.

### 2.6. *β*-Galactosidase Senescence Staining

For antisenescence experiments, human endothelial cells were passaged from the 10th or 15th generation to the 40th or 45th generation and cultured with or without bavachalcone in DMEM containing 10% serum. In a part of the experiment, HUVECs cultured with ROR*α* receptor inhibitor VPR-66 (NBP2-29335, Novus Biologicals, Littleton, CO, USA) were used. For senescence staining, the cellular senescence assay kit (Cell Biolabs, Inc., San Diego, CA, USA) was used. Rat aortal tissue and HUVECs were fixed in 1% formaldehyde containing 0.2% glutaraldehyde in PBS for 5 min at room temperature, rinsed with PBS, and incubated at 37°C with a fresh *β*-galactosidase stain solution: 1 mg/mL 5-bromo-4-chloro-3-indolyl *β*-D galactoside, 40 mmol/L citric acid/sodium phosphate (pH 6.0), 5 mmol/L potassium ferrocyanide, 150 mmol/L NaCl, and 2 mmol/L MgCl_2_.

### 2.7. Statistical Analysis

Data are expressed as the mean ± standard deviation (SD). Paired *t*-test analysis and one-way ANOVA were used to compare the differences between and within the groups. All statistical analyses were performed using SPSS Version 15.0 or GraphPad Prism 5. *P* value less than 0.05 was considered statistically significant.

## 3. Results

**Figure 1:**
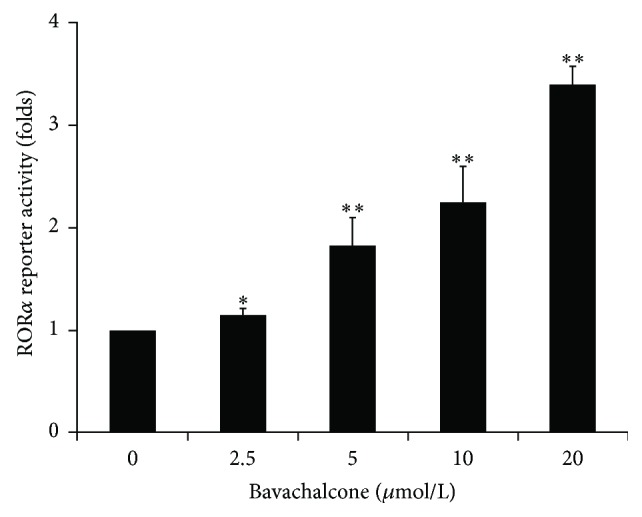
Bavachalcone-activated ROR*α* reporter luciferase activity. Transient transfected HEK293 cells containing ROR*α* (3× RORE) reporter luciferase plasmids were stimulated with the indicated doses of bavachalcone for 16 h. The cells were lysed and analyzed for luciferase activity (*n* = 3). Data are expressed as the mean ± SD. ^*∗*^ *P* < 0.05 versus vehicle control; ^*∗∗*^ *P* < 0.01 versus vehicle control.

**Figure 2:**
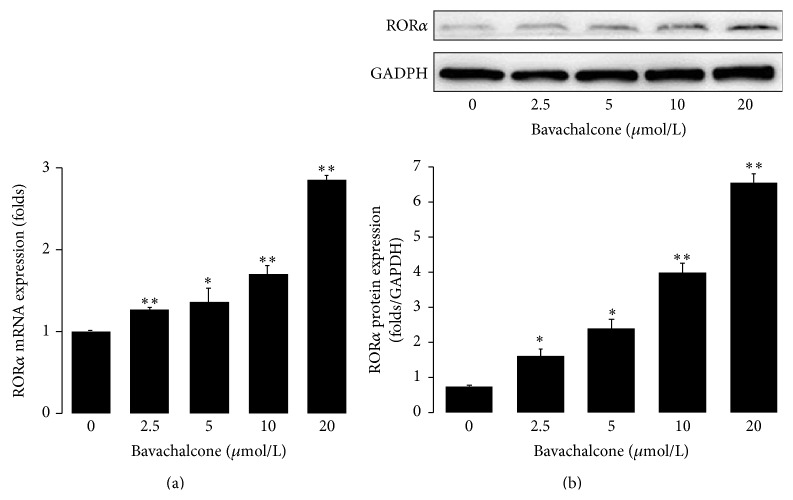
Bavachalcone induced ROR*α* expression. HUVECs were treated with 20 *μ*mol/L of bavachalcone for 24 h, and mRNA (a) and protein (b) expression levels were measured using real-time quantitative PCR or western blotting (*n* = 3 each). Data are expressed as the mean ± SD. ^*∗*^ *P* < 0.05 versus vehicle control; ^*∗∗*^ *P* < 0.01 versus vehicle control.

**Figure 3:**
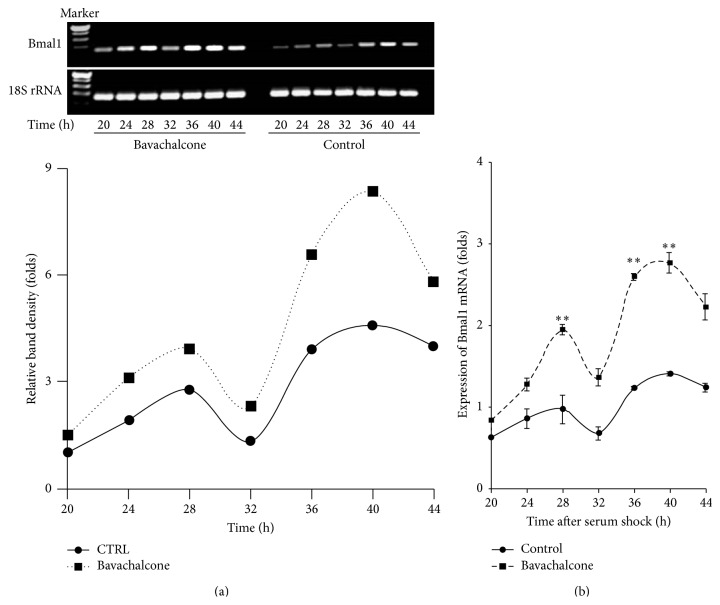
Bavachalcone-enlarged expression amplitude of the circadian gene Bmal1. After HUVECs were treated with 50% serum shock for 1 h, the medium was replaced with 10% serum DMEM with or without 20 *μ*mol/L bavachalcone. The mRNA expression levels at the indicated time were measured using semiquantitative (a) and real-time quantitative PCR (b) (*n* = 3 each). Data are expressed as the mean ± SD. ^*∗∗*^ *P* < 0.01 versus vehicle control.

*Bavachalcone Induces RORα1 Expression and Regulates Bmal1 Circadian Rhythm*. In the screening of natural ingredient activity, bavachalcone exhibited characteristics that can activate ROR*α*1 expression (Table 1). Bavachalcone's dose effect relationship showed that it dose-dependently activated ROR*α*1 luciferase reporter gene activity, causing a more than 3-fold increase at a dose of 20 *μ*mol/L (*n* = 3, *P* < 0.01, Figure 1). Moreover, bavachalcone dose-dependently induced ROR*α*1 expression in mRNA and protein levels, causing a more than 2-fold increase in mRNA expression (*n* = 3, *P* < 0.01, Figure 2(a)) and a 6-fold increase in protein expression (*n* = 3, *P* < 0.01, Figure 2(b)) at a dose of 20 *μ*mol/L. Furthermore, bavachalcone dynamically regulated circadian gene Bmal1 mRNA expression, a ROR*α*1 downstream signal. As shown in Figures 3(a) and 3(b), from 24 to 48 h after 50% serum shock, treatment with bavachalcone enhanced the circadian amplitude of Bmal1 mRNA expression on 28, 36, and 40 h, which was far wider than other time points compared with the control, with the differences being significant (*n* = 3, *P* < 0.01) at each time point. 

**Figure 4:**
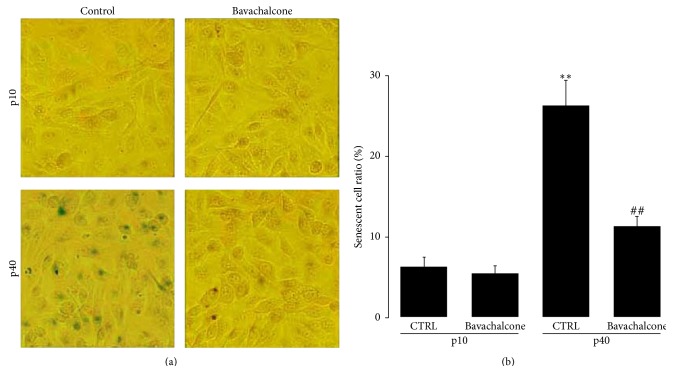
Bavachalcone-delayed senescence of HUVEC. (a and b) HUVECs were passaged from the 10th to the 40th generation and cultured with or without bavachalcone. Subsequently, the cells were dyed using a *β*-galactosidase senescence assay kit. The percentage of senescent cells was obtained by counting more than 500 cells in each sample (*n* = 5 each). Data are expressed as the mean ± SD. ^*∗∗*^ *P* < 0.01 versus p10 passage vehicle control. ^##^ *P* < 0.01 versus p40 passage vehicle control.

**Figure 5:**
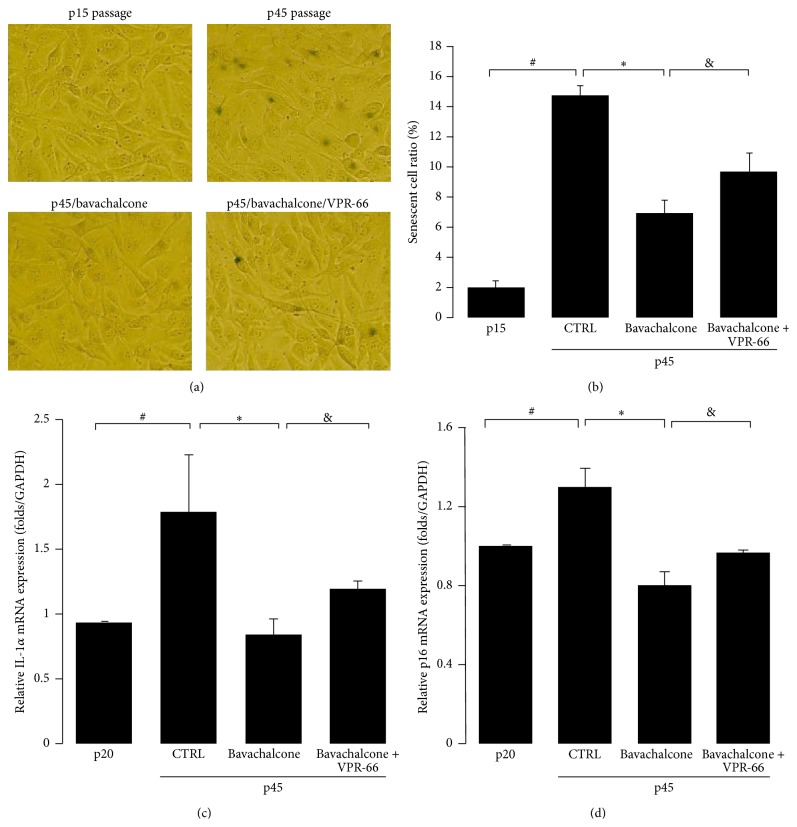
Bavachalcone-delayed cellular senescence, partially through ROR*α*-Bmal1 pathway. (a and b) HUVECs were passaged from the 15th to the 45th generation and treated with or without bavachalcone and with or without VPR-66, a ROR*α* inhibitor. Subsequently, the cells were dyed using a *β*-galactosidase senescence assay kit. The percentage of senescent cells was obtained by counting more than 500 cells in each sample (*n* = 5 each). (c and d) HUVECs were passaged from the 20th to the 45th generation and treated with or without bavachalcone and with or without VPR-66. The mRNA expression levels were measured using real-time quantitative PCR (*n* = 3 each). Data are expressed as the mean ± SD. ^#^ *P* < 0.01 versus p15 or p20 passages vehicle control; ^*∗*^ *P* < 0.01 versus p45 passage vehicle control; ^&^ *P* < 0.05 versus p45 passage bavachalcone treatment.

*Bavachalcone Suppressed Replicative Senescence of Endothelial Cells Partially through RORα1-Bmal1 Pathway*. Bavachalcone saliently delayed the replicative senescence of endothelial cells; bavachalcone treatment reduced the senescent cell ratio from 26.4% ± 3.0% to 11.3% ± 1.2% at the p40 passage (*n* = 5, *P* < 0.01, Figures 4(a) and 4(b)). Subsequently, to determine the relationship between cellular senescence and ROR*α*, endothelial cells were incubated with the ROR*α* inhibitor VPR-66 for 24 h. The results revealed that VPR-66 partially antagonized the antiaging effect of bavachalcone (*n* = 5, *P* < 0.05, Figures 5(a) and 5(b)). To clearly define the status of cellular senescence, the mRNA expression of p16^ink4a^ and IL-1*α* was determined. Vascular endothelial cells undergoing replicative senescence exhibited increased expression of p16^ink4a^ and IL-1*α* mRNA, but their expression was significantly suppressed by bavachalcone (*n* = 3 each, *P* < 0.01, Figures 5(c) and 5(d)). However, coincubation with the ROR*α* inhibitor VPR-66 for 24 h partially reversed the inhibitory effect of bavachalcone on the mRNA expressions of p16^ink4a^ and IL-1*α* (*n* = 3, *P* < 0.05, Figures 5(c) and 5(d)).

## 4. Discussion

ROR*α* is a member of the ROR family, and its ROR*α* function has been implicated in several pathological processes, such as cancer, autoimmune diseases, inflammation, osteoporosis, and metabolic syndrome [16]. Our results show that bavachalcone activates ROR*α*1 luciferase reporter activity and mRNA and protein expression. Our previous findings show that bavachalcone activates AMPK activity and induces MnSOD expression [14]. In addition, ROR*α*1 activates AMPK activity [17, 18], and reduces oxidative stress through the induction of MnSOD and GPx1 expression [9]; our results are consistent with these reports. Numerous studies have indicated that the transcriptional activating function of ROR*α*1 is regulated not only by its natural ligands, such as cholesterol and cholesterol derivatives, but also by synthetic ligands [16].

Our results show that bavachalcone delays cell senescence and inhibits mRNA expression of p16^ink4a^ (a maker of replicative senescence [19]) and IL-1*α* (a proinflammatory cytokine of the senescence-associated secretory phenotype [20]). These inhibitory effects could be partially reversed by the ROR*α* inhibitor VPR-66. The Bmal1 promoter region contains ROR response elements where the ROR families can bind [21], supporting our results that the dynamic expression of Bmal1 gene is regulated by bavachalcone-enhanced ROR*α*1 activity. Studies have reported that deficiency of Bmal1 increases vascular superoxide production; endothelial nitric oxide synthase uncoupling; and COX-2, Nox4, MMP2, and MMP9 expressions and underlies vascular stiffness [22–24]. In addition, studies have shown that deficiency of the circadian clock transcriptional factor BMAL1 impairs glucose homeostasis, exhibits premature age-associated disorders, reduces lifespan [25–27], and increases sensitivity to genotoxic stress [28]. A recent study reported that reactive oxygen species exhibits a wave in the circadian rhythm in the brains of wild-type mice, but not in Bmal1 deficient mice [29]. In addition, deficiency of clock gene, a Bmal1 partner, reduces lifespan and increases age-related characteristics [30].

In conclusion, through a luciferase assay, we showed that bavachalcone induced ROR*α*1 expressions at luciferase reporter, mRNA, and protein levels in human endothelial cells and dynamically regulated Bmal1 mRNA expression. In addition, bavachalcone suppressed replicative senescence in human endothelial cells partially through the ROR*α*-Bmal1 pathway. Our results demonstrate that bavachalcone, a natural medicine ingredient, has a pharmacological function in regulating ROR*α*.

## Figures and Tables

**Table 1:** Screening list using ROR*α*1 luciferase reporter (dose = 10 *μ*mol/L).

Chemical names	Chemical structures	CAS number	Molecular weight	Expression (folds/control, *n* = 3, mean ± SD)
Bavachalcone	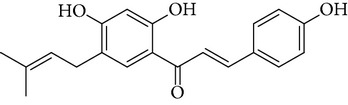	28448-85-3	324.4	2.2295 ± 0.0785

Isobavachalcone	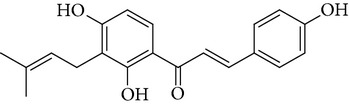	20784-50-3	324.4	0.9188 ± 0.1373

Bavachin	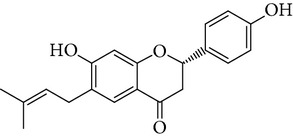	19879-32-4	324.4	1.0106 ± 0.0721

Isobavachin	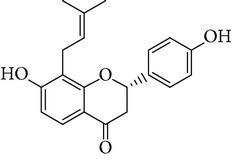	31524-62-6	324.4	0.9988 ± 0.0472

Glabranin	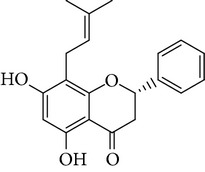	41983-91-9	324.4	1.0901 ± 0.0405

Mulberrin	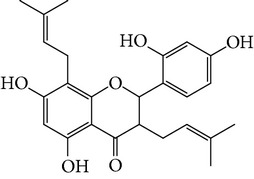	62949-79-5	422.5	1.2416 ± 0.1792

Psoralidin	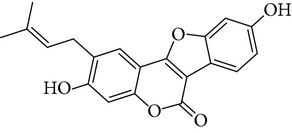	18642-23-4	336.3	0.9612 ± 0.0415

Icariin	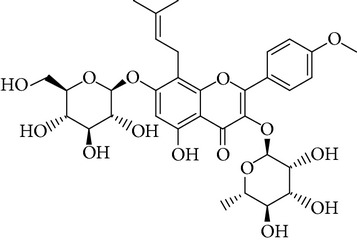	489-32-7	676.7	1.2514 ± 0.2206

Corylin	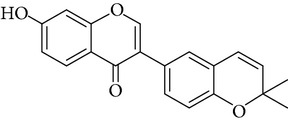	53947-92-5	320.3	0.9953 ± 0.0188

Olivil	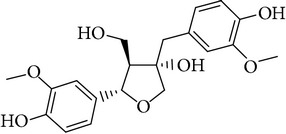	2955-23-9	376.4	0.9471 ± 0.1459

Psoralen	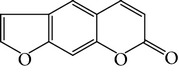	66-97-7	186.2	1.0530 ± 0.0497

Angelicin	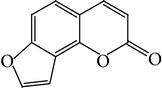	523-50-2	186.2	0.9476 ± 0.0542

8-Methoxypsoralen	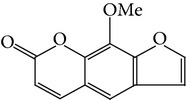	298-81-7	216.2	1.0068 ± 0.0182

Pinoresinol diglucoside	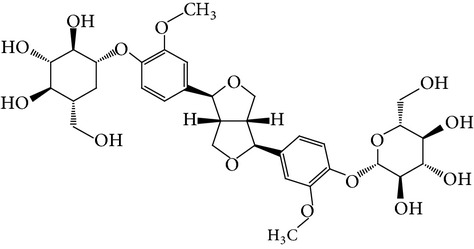	63902-38-5	682.7	1.1309 ± 0.0533

Eleutheroside E	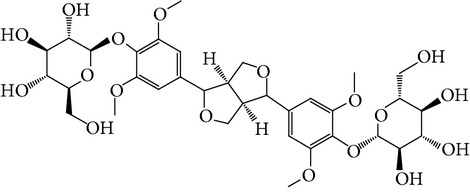	39432-56-9	742.7	1.0357 ± 0.0305

Catechin	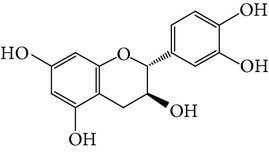	7295-85-4	290.3	1.0800 ± 0.0681

Coniferin	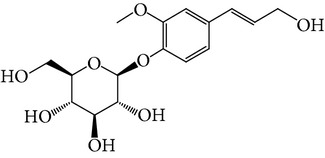	531-29-3	342.3	1.3680 ± 0.1221

Genipin	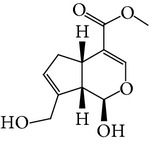	6902-77-8	226.2	1.0714 ± 0.0847

Geniposide	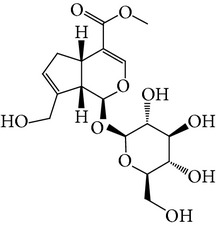	24512-63-8	388.4	0.9631 ± 0.0851

Aucubin	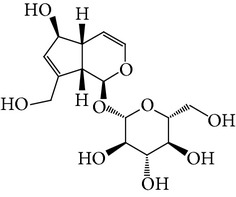	479-98-1	346.3	0.9624 ± 0.2046
